# Analysis of K_ATP_ Channels Opening Probability of Hippocampus Cells Treated with Kainic Acid

**DOI:** 10.21315/mjms2021.28.1.3

**Published:** 2021-02-24

**Authors:** Mohd Harizal Senik, Izuddin Fahmy Abu, Widad Fadhullah

**Affiliations:** 1 Department of Neurosciences, School of Medical Sciences, Universiti Sains Malaysia, Kelantan, Malaysia; 2 School of Life Sciences, Medical School, Queen’s Medical Centre, University of Nottingham, Nottingham, United Kingdom; 3 Institute of Medical Science Technology, Universiti Kuala Lumpur, Kuala Lumpur, Malaysia; 4 School of Industrial Technology, Universiti Sains Malaysia, Pulau Pinang, Malaysia

**Keywords:** channel opening probability, epilepsy, hippocampus cells, Kainic acid, K_ATP_ channels, pancreatic beta-cells, potassium channel

## Abstract

**Background:**

Kainic acid (KA)-induced seizures may be a valuable tool in the assessment of anti-epileptic drug efficacy in complex partial seizures. This study investigated the effects of KA on ATP-sensitive K^+^ (K_ATP_) channels opening probability (NPo), which plays a crucial role in neuronal activities.

**Methods:**

For the optimisation and validation protocol, β-cells were plated onto 35 mm plastic petri dishes and maintained in RPMI-1640 media supplemented with 10 mM glucose, 10% FCS and 25 mM of N-2-hydroxyethylpiperazine-N-ethanesulfonic acid (HEPES). The treatment effects of 10 mM glucose and 30 μM fluoxetine on K_ATP_ channels NPo of β–cells were assessed via cell-attached patch-clamp recordings. For hippocampus cell experiments, hippocampi were harvested from day 17 of maternal Lister-hooded rat foetus, and then transferred to a Ca^2+^ and Mg^2+^-free HEPES-buffered Hank’s salt solution (HHSS). The dissociated cells were cultured and plated onto a 25 mm round cover glasses coated with poly-d-lysine (0.1 mg/mL) in a petri dish. The K_ATP_ channels NPo of hippocampus cells when perfused with 1 mM and 10 mM of KA were determined.

**Results:**

NPo of β-cells showed significant decreasing patterns (*P* < 0.001) when treated with 10 mM glucose 0.048 (0.027) as well as 30 μM fluoxetine 0.190 (0.141) as compared to basal counterpart. In hippocampus cell experiment, a significant increase (*P* < 0.001) in mean NPo 2.148 (0.175) of neurons when applied with 1 mM of KA as compared to basal was observed.

**Conclusion:**

The two concentrations of KA used in the study exerted contrasting effects toward the mean of NPo. It is hypothesised that KA at lower concentration (1 mM) opens more K_ATP_ channels, leading to hyperpolarisation of the neurons, which may prevent neuronal hyper excitability. No effect was shown in 10 mM KA treatment, suggesting that only lower than 10 mM KA produced significant changes in K_ATP_ channels. This implies further validation of KA concentration to be used in the future.

## Introduction

Epileptogenesis is the process of developing epilepsy, characterised by seizures that not only impair quality of life but can also lead to mortality (1). Epilepsy is normally caused by the imbalance of excitatory and inhibitory neurotransmitters in the brain, where glutamate is the predominant excitatory neurotransmitter in the central nervous system (2–4). Glutamatergic transmission plays a key role in the brain; however, overstimulation of glutamate receptors (GluRs) may lead to excitotoxicity (5, 6). Kainic acid (KA) is one of the excitatory amino acid that may stimulate GluRs (7). Experimentally, KA was frequently used to induce temporal lobe epilepsy (TLE) in animals (8–11) as a model to mimic human TLE. KA administration in rats can replicate three features of TLE by inducing initial injury, which affects the hippocampus and/or the temporal lobe (12), creating a latent period between the damage and causing the occurrence of spontaneous seizures (9, 10, 13). Previous studies had shown that local administration (injected directly into the brain) or systemic administration (via intra-peritoneal injection) of KA in rodents triggered repetitive limbic seizures, status epilepticus (SE) (8, 14, 15) and caused neuronal degeneration of the selected population of neurons in the brain (16).

Four different classes of K^+^ channels have been identified: i) voltage-dependent K^+^ channels (K_V_); ii) Ca^2+^-activated K^+^ (K_Ca_) channels; iii) ATP-sensitive K^+^ (K_ATP_) channels and iv) inward rectifier K^+^ (K_ir_) channels (17, 18). However, K_ATP_ channels have been placed under the K_ir_ super family of K^+^ channels as it conducts weak inward rectifier potassium current (19, 20). K^+^ channels are the most dominant ion conductive pathways in electrically excitable cells and regulate neuronal excitability by controlling the firing frequency of the action potentials (20, 21). The opening of the K^+^ channels hyperpolarise the cell by causing an efflux of K^+^ ions. Subsequently, this condition is followed by the closure of voltage-dependent Ca^2+^ channels, with the consequent reduction in Ca^2+^ entry and vasodilatation (22, 23). On the other hand, the closure of K^+^ channels causes membrane depolarisation and vasoconstriction (23–25). Thus, in the presence of a physiological or pharmacological agent that alters membrane potential, for instance, the K_ATP_ channel openers cromakalim and pinacidil, may cause a significant change in blood vasolidation (22, 26, 27).

K_ATP_ channels are ubiquitously present in cells, such as myocytes, pancreatic beta (β)-cells, and neurons (20, 28). K_ATP_ channels comprise of four pore-forming subunits (K_ir_6.1 or K_ir_6.2 encoded by KCNJ8 and KCNJ11, respectively) and four regulatory sulfonylurea receptors (SUR) ATP-binding cassettes subunits (subfamily C: SUR1, SUR2A or SUR2B) (20). K_ATP_ channels have been implicated in models of tissue injury, including the heart and brain (29). For example, a mutation in K_ir_6.2 results in a syndrome of developmental delay, infantile diabetes, and epilepsy (30, 31). The association of K_ir_6.2 channels and epilepsy has been previously described to be mainly caused by KCNJ11 mutation-related subtypes as demonstrated in diabetes with epilepsy as a co-morbidity (31–33). In diabetic conditions, K_ATP_ channels in the β-cells of the pancreas exert an anti-hyperglycaemic effect by stimulating insulin secretion due to their closure in response to increasing plasma glucose concentration (34). Sulfonylureas are used in the treatment of non-insulin-dependent diabetes mellitus as oral hypoglycaemic agents by closing the K_ATP_ channels. The binding of sulfonylurea to the SUR induces closure of the channels and results in membrane depolarisation of the pancreatic β-cells, which in turn stimulates the secretion of insulin (35).

K_ATP_ channels have been reported to demonstrate a significant role in neuroprotection and treating epileptic disorders in diabetic hyperglycaemia (36) and neuronal excitability in metabolic stress (19, 29, 37). This mechanism initiates a more excitable state, which implies that an increase in extracellular glucose and intracellular ATP decreases K_ATP_ channels (36). Apart from regulating neuronal excitability, neuronal K_ATP_ channels also play an essential role in spontaneous firing in various neurons, including cholinergic basal forebrain neurons, expiratory neurons, entorhinal layer three cortical neurons, substantia nigra neurons, and thalamocortical neurons. Activation of K_ATP_ channels have also been shown to be neuroprotective in both focal and global ischemia as evident in in vivo models as well as in vitro experiments. These studies suggest that the effects are mediated at least in part by neuronal K_ATP_ channels (20). K_ATP_ channels were demonstrated to regulate the release of neurotransmitters and are involved in the protection against glutamate excitotoxicity in in vivo and cultured hippocampal neurons (28). K_ATP_ channels are activated by Mg-ADP and blocked by ATP; these channel characteristics allow the cells to couple cellular metabolic state (ATP/ADP ratio) to electrical activity of the cell membrane (20, 38). In a way, the connection between the level of electrical activity and intracellular ATP concentration may suggest that K_ATP_ channel through its regulation of neuronal excitability may also serve for a potential anti-epileptic mechanism (36).

Since K_ATP_ channels are essentially implicated in neuronal activities such as neurotransmission, it is postulated that they play a fundamental role in the underlying process of direct or indirect neuronal hyper excitability. Consequently, this resulting epilepsy may lead to excitotoxic cell death. Therefore, this study aimed to provide preliminary K_ATP_ profiling of hippocampus cells treated with KA.

## Methods

In this study, β–cells were used to optimise and validate the methods. K_ATP_ channels activity of β-cell-line was investigated using a single channel patch-clamp technique in the cell-attached configuration. Hippocampus cells were later used after β–cell validation and optimisation using the same protocol.

Considering the important role of pancreatic K_ATP_ channels in regulating the secretion of insulin, this study investigated K_ATP_ channel activity in the presence of glucose and fluoxetine. Fluoxetine is a Food and Drug Administration (FDA)-approved antidepressant belonging to selective serotonin reuptake inhibitors (SSRI) class and used for the treatment of major depressive disorder, obsessive-compulsive disorder, bulimia nervosa and panic disorder. This antidepressant drug has been previously reported to interfere with blood glucose levels in rodents (39, 40), presumably by the interaction of K_ATP_ channels to modulate channel activity.

### Preparation of β-cells (Optimising and Validating Purposes)

β-cells were plated onto 35 mm plastic petri dishes (Nunc). Cells were cultured and maintained in RPMI-1640 media supplemented with 10 mM glucose, 10% FCS and 25 mM HEPES. The petri dishes were then kept in humidified air/5% CO_2_ at normal body temperature (37 °C). The concentration of 10 mM glucose was chosen to mimic the physiological blood glucose concentration within the range of 5 mM to 8 mM in subjects possessing a healthy metabolism and 11 mM in diabetes patients after glucose exposure (41).

### Preparation of Hippocampus Cells

**Figure 1:**
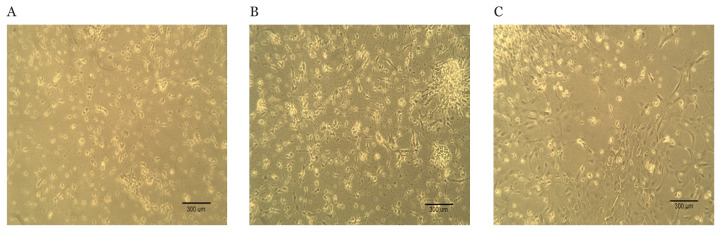
Hippocampus cell culture for cell-attached experiments at **A**. day 1 **B**. day 7 and **C**. day 14

All procedures were carried out in accordance with the Animals (Scientific Procedures) Act 1986, UK and have been approved by the UK Home Office (project license 40/3283 and personal license 40/10438). Maternal Lister-hooded rats at embryonic day 17 were anaesthetised with CO_2_ and sacrificed by decapitation to collect the foetuses (42). Hippocampi were harvested and transferred to a Ca^2+^ and Mg^2+^-free HEPES-buffered Hank’s salt solution (HHSS), pH 7.45. Trituration was performed to dissociate the cells, which then were pelleted and re-suspended in Dulbecco’s Modified Eagle’s Media (DMEM) without glutamine and supplemented with 10% foetal bovine serum (FBS), penicillin (100 U/mL) and streptomycin (100 μg/mL). The dissociated cells were then plated onto a 25 mm round cover glasses coated with poly-d-lysine (0.1 mg/mL) at a density of 50000 cells/well and washed with distilled water. The resulting neuron cells were cultured in DMEM containing 10% FBS and penicillin/streptomycin for 24 h in a humidified atmosphere with 10% CO_2_ and 90% air (pH 7.4) at 37 °C. The cells were fed every 7 days by replacing 70% of the media with DMEM supplemented with 10% horse serum and penicillin/streptomycin. The cells used in the above protocol were cultured without mitotic inhibitors for a minimum of 12 days (42, 43). The cells were ready to use on day 14. Hippocampus cell culture images for cell-attached experiments at day 1, 7 and 14 are shown in Figure 1.

### Solutions

In the β-cells attached patch experiments for the measurement of K_ATP_ current; the cells were pre-incubated prior to use in a sugar-free Hank’s solution for 20 min–30 min at 37 °C. Experiments were performed in a high K^+^ HEPES-buffered Hanks’ salt solution (HHSS) containing (in mM) 138 NaCl, 4.2 NaHCO_3_, 1.2 NaH_2_PO_4_, 5.6 KCl, 1.2 MgCl_2_, 2.6 CaCl_2_, 10 HEPES (pH 7.4 with NaOH) as adapted from Smith et al. (44). To study the effect of glucose and fluoxetine on β–cell for the optimisation and validation protocol, 10 mM glucose and 30 μM fluoxetine was added to a high potassium depolarising (HiK) solution in two different beakers, respectively.

To study KA-treated hippocampus cells, two different concentrations of KA (1 mM and 10 mM) were added separately to HHSS solution that contained i) 20 mM HEPES; ii) 137 mM NaCI; iii) 1.3 mM CaCl_2_; iv) 0.4 mM MgSO_4_; v) 0.5 mM MgCI_2_; vi) 5.0 mM KCI; vii) 0.4 mM KH_2_PO_4_; viii) 0.6 mM Na_2_HPO_4_; ix) 3.0 mM NaHCO_3_ and x) 5.6 mM glucose.

For cell-attached patch-clamp recording, the pipette solution consisting of the followings (in mM): i) 140 KCl; ii) 2.6 CaCl_2_; iii) 1.2 MgCl and iv) HEPES (pH 7.4) was prepared.

### Cell-Attached Patch-Clamp Recordings

Borosilicate glass capillary pipettes (GC150TF-15; Harvard, United Kingdom) pulled with a 2-stage vertical putter (Narishige PP83, Japan) were used for the patch-clamp recordings. Bubble number 7 was used to determine the diameter of the pipette tip. Sylgard silicone elastomer (Dow Corning Corp, Michigan, USA) was applied below the pipette tips and subsequently fire-polished before the use to avoid electrical noise. The pipettes were filled with a HiK solution using a micro-syringe. Any bubbles from the tip were removed with extra care. The suitable standard pipette resistances for use were between 2.5 MΩ and 5 MΩ.

**Figure 2:**
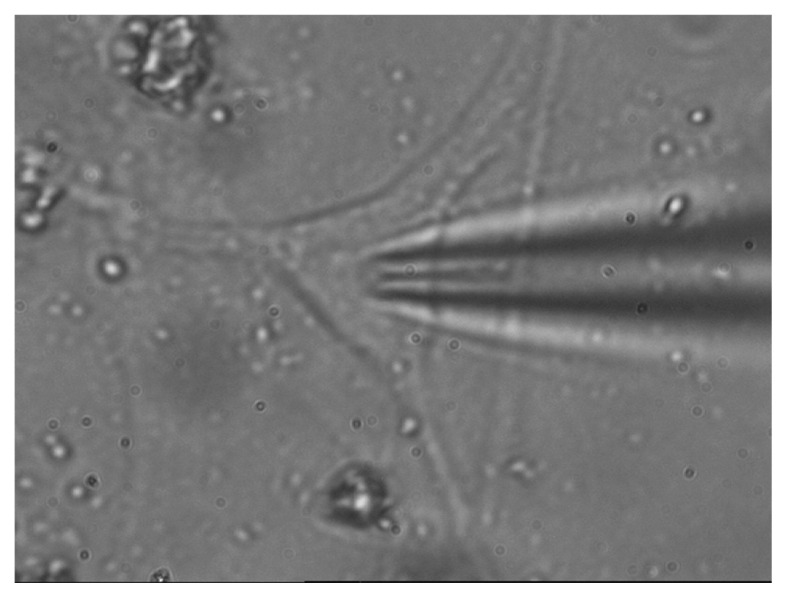
Position of hippocampus cell and micropipette for cell-attached experiment

Prior to the patching process, the β-and hippocampus cells were washed using Hank’s solution. Only cells that were evidently round and morphologically distinct were patched. The patch was sealed by pressing the pipette against the cell surface before applying light suction to the inside of the pipette. The position of hippocampus cell and micropipette for the cell-attached experiment is demonstrated in Figure 2. Only patches forming seals in the giga-ohm range were recorded.

The cell-attached recordings were made using an Axopatch patch-clamp amplifier (Molecular Devices Inc., Sunnyvale, CA, USA). Single-channel currents were filtered at 2 kHz prior to digitisation at 10 kHz using Clampfit 10.3 (Molecular Devices Inc., Sunnyvale, CA, USA) connected to Axopatch-1D patch-clamp amplifier (Axon instruments, San Jose, CA, USA). Single-channel currents were recorded at a pipette potential of 0 mV, at the resting membrane potential (44). All procedures were conducted within room temperature of 21 °C–23 °C.

The channel activities were measured by the open probability (NPo). All NPo values were calculated from 3 min of a single-channel recording. Data from each patch membrane served as the control. NPo after seal and perfusion by Hank’s solution served as the basal record and was compared with NPo of treatment as adapted from Smith et al. (44).

### Statistical Analysis

One-way ANOVA was performed followed by Bonferroni’s multiple comparison tests using Graphpad Prism 8.0 to determine the statistical significance between glucose, fluoxetine and basal in the β-cell experiment and between two concentrations of KA (1 mM and 10 mM) and basal in the hippocampus cell experiment. *P-*values of less than 0.001 were considered statistically significant. The data were presented as mean (SEM), where *n* is the number of cell-attachment experiments and each ones were from a different petri dish.

## Results

### Optimisation and Validation Experiment Using β-Cells

**Figure 3:**
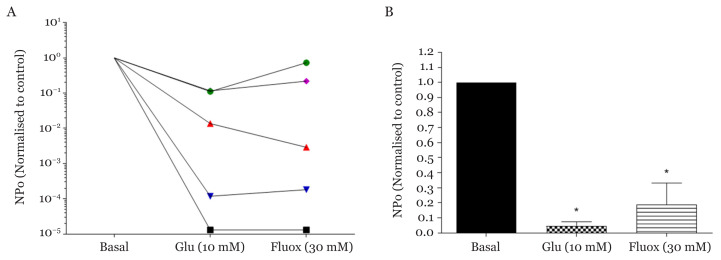
Effects of glucose and fluoxetine on single K_ATP_ channels opening probability (NPo) in 3 min perfusion (*n* = 5). **A**. Individual experiment (represented by each line) showed reduced NPo of K_ATP_ channels when applied with 10 mM of glucose and 30 μM fluoxetine compared to basal counterpart (HiK solution). **B**. Mean NPo ratio of K_ATP_ channels activity when perfused with glucose and fluoxetine in comparison to basal. NPo of glucose and fluoxetine are normalised to basal, respectively *Statistically significant difference with comparison to basal, *P* < 0.001 using one-way ANOVA followed by Bonferroni’s multiple comparisons test

All individual experiments (*n* = 5) showed similar patterns with lower K_ATP_ channels NPo in β-cells when applied with 10 mM glucose as well as 30 μM fluoxetine and being normalised to basal record (HIK solution) as the control (Figure 3A). Figure 3B, shows a significant decrease of NPo of K_ATP_ channels activity when applied with glucose 0.048 (0.027) and fluoxetine 0.190 (0.141) compared to basal (*F*_(2, 12)_ = 38.26, *P* < 0.001; one-way ANOVA). The single-channel K_ATP_ blockage through the addition of 10 mM glucose was demonstrated by the drop of mean NPo, in agreement with previous findings by Tringham et al. (45). The reason for this phenomenon is due to the concurrent surge in cell metabolism and increased ATP/ADP ratio, which inhibits the K_ATP_ channels.

### Hippocampus Cell-Attached Experiment

**Figure 4:**
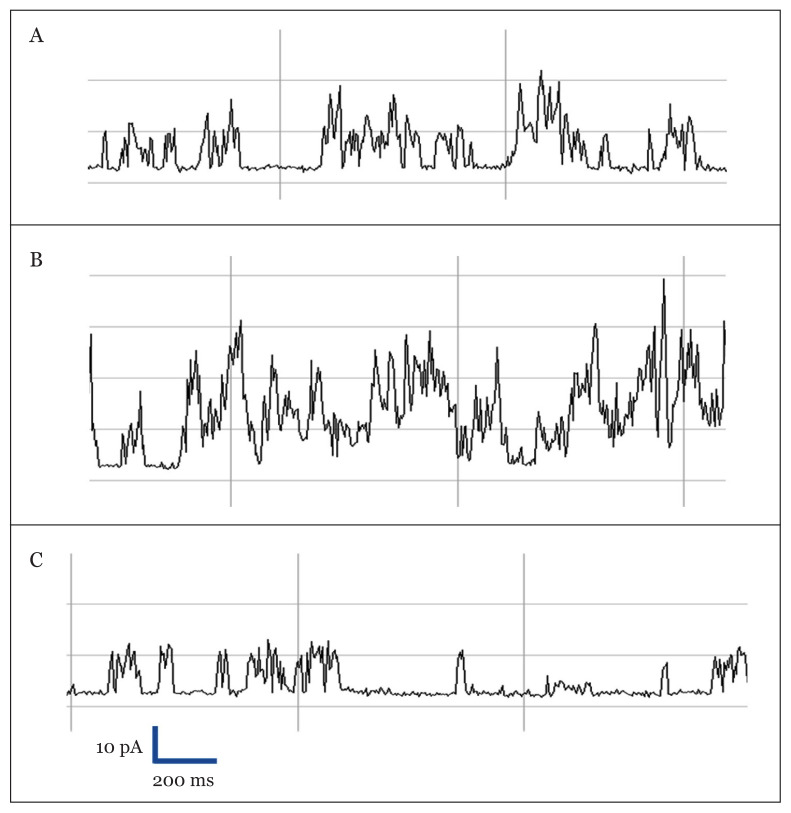
Representative records of K_ATP_ activity measured in a hippocampus cell-attached patch with a pipette potential of +70 mV. The images show K_ATP_ single-channel currents during **A**. perfusion of unmodified HHSS solution (basal), **B**. in the presence of 1 mM of KA in HHSS, and **C**. in the presence of 10 mM of KA in HHSS

As this observation is dependent on cell conditions, therefore, it was important to use the cell-attached configuration to retain the KA metabolism of the hippocampus cells intact. In order to characterise the K_ATP_ single-channel activities in cell-attached, the channel currents were made and visually evaluated. Representative results of the effects of 1 mM and 10 mM KA on K_ATP_ single-channel activities are depicted in Figure 4. Figure 4A shows the single-channel records under unmodified condition of HHSS bath solution while Figure 4B, displays the reactivation of the K_ATP_ channels after the addition of 1 mM KA in HHSS solution. Figure 4C, demonstrates the subsequent bath application of 10 mM KA in HHSS solution.

**Figure 5:**
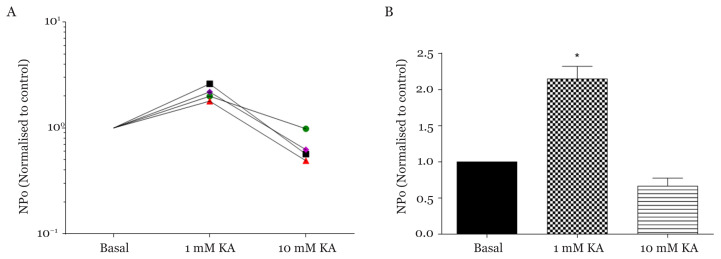
Effects of KA in two different concentrations (1 mM and 10 mM) on K_ATP_ channels activities in 3 min perfusion (*n* = 4). **A**. Effects of KA on K_ATP_ channels activities (NPo) during 3 min interval as shown by individual experiments. **B**. Effects of KA on NPo ratio of K_ATP_ channels activities in comparison to basal. NPo of 1 mM and 10 mM of KA are normalised to basal (HHSS solution), respectively *Statistically significant difference in comparison to basal, *P* < 0.001 using one-way ANOVA followed by Bonferroni’s multiple comparisons test

As shown in Figure 5A, it was observed that the two concentrations of KA used in this study gave two different effects on the NPo of K_ATP_ channels. All individual experiments (*n* = 4) demonstrated that 1 mM KA increased the NPo compared to basal. The ratio of NPo when perfused with 1 mM KA was significantly increased 2.148 (0.1748) as compared to basal (HHSS solution) (*F*_(2, 9)_ = 42.72, *P* < 0.001) (Figure 5B). In contrast, the difference of NPo ratio between KA at a higher concentration (10 mM) and basal was not statistically significant. It is hypothesised that 1 mM KA opens more K_ATP_ channels and lead to the hyper polarisation of the neurons.

## Discussion

In our study utilising pancreatic β-cell, K_ATP_ channels were shown to be blocked by 10 mM glucose which is consistent with early documented reports on the effects of glucose on K_ATP_ channel kinetics (46–48). This is due to the concomitant elevation in cell metabolism and the increase of ATP/ADP ratio, which inhibits the K_ATP_ channels. Studies have shown that when plasma glucose level rises in pancreatic β-cells, the concentration of ATP increases the glucose level, which results in closure of K_ATP_ channels (49, 50). K_ATP_ channels closure in the plasma membrane allows the cells to depolarise, subsequently triggering calcium entry and insulin release (51–53).

In this experiment, 30 μM fluoxetine also caused blockage of K_ATP_ channels as shown by the decrease of NPo. Fluoxetine is currently an important drug for the treatment of major depression and obsessive-compulsive disorder. Previous and recent studies have reported that depression is a critical problem in diabetic patients (54–57). It has also been documented that the fluoxetine elevates blood glucose and reduces plasma insulin levels (40, 54, 58–60). In the present study, the effect of glucose and fluoxetine was examined in the context of the insulin-secretory pathway in the pancreatic β-cells. They were also used to optimise and validate the method and to compare the K_ATP_ profile.

The hippocampus is among the most vulnerable region towards KA-induced neuronal death (61, 62). KA induces continuous depolarisation and high intracellular Ca^2+^ entry, which potentiates the release and action of endogenous excitatory amino acids, activation of caspases, and nitric oxide production. These actions initiate excitotoxic cell death by necrosis, apoptosis, or both (63–67). In this preliminary study, the findings demonstrated two varied effects from two different KA concentrations (1 mM and 10 mM). Lower concentration of KA (1 mM) significantly increased NPo of K_ATP_ channel of neurons during the 3 min acute perfusion that may generally cause neuronal hyper polarisation. K_ATP_ openers mimic some of the neuroprotective effects of preconditioning, and in contrast, K_ATP_ blockers were used to reverse these effects (61). The mechanism involved suggests that activation of K_ATP_ channels through adenosine receptors may act as an early step of ischemic-cerebral preconditioning (20, 68, 69). K_ATP_ channels are regulated by the ATP/ ADP ratio in a way that a drop of this ratio will activate these channels. Following their opening, the efflux of K^+^ will induce a hyper polarisation and decreases the neuronal excitability. K_ATP_ channels also control neurotransmitter release by regulating neuronal excitability (38). K_ATP_ channels may down regulate glutamate release (28, 70), and their over expression has beneficial effects in epilepsy (38, 71) and schizophrenia (72).

The K_ATP_ channels in the hippocampus are thought to possess neuroprotective roles; cellular stress will activate these channels causing a transient membrane hyper polarisation with a consequent decrease of energy demand. Thus, rendering sufficient protection to the metabolically compromised cells (38). The K_ATP_ channels are coupled to the intracellular energy supply; when ATP levels are high, K_ATP_ channels are closed, whereas during prolonged action potential firing K_ATP_ channels will eventually contribute to resetting (31, 73). Seizure-like activity in hippocampal slices may be induced by Mg^2+^-free solution accompanied by ATP decrease and activation of K_ATP_ channels. Increased ATP consumption may contribute to excitotoxicity (74). In our case, 1 mM of KA resulted in increased patterns of NPo which may cause hyper polarisation in hippocampus cells. By acting via membrane hyper polarisation and preventing propagation of the excitatory postsynaptic current, this mechanism may protect neurons from potentially excitotoxic insults (28). Simulations by Krishnan and Bazhenov (75) proposed significant effects of sodium accumulation in termination of seizures where, K_ATP_ channels would close the loop: GluR**→**Na^2+^**→**Na^2+^/K^+^-ATPase**→**ATP/ADP.

## Conclusion

This study showed contrasting effects of NPo between pancreatic β-cells and hippocampus cells. In the presence of glucose and fluoxetine, NPo of K_ATP_ channels in β-cells decreased, while hippocampus cells treated with 1 mM of KA showed increased NPo of K_ATP_ channels. Despite the small sample size, this study provided important preliminary information towards reaching a concrete conclusion on the effects of KA towards K_ATP_ channels activities in hippocampus cells. As this study unexpectedly saw a wide contrasting effect in K_ATP_ profiling between the two KA concentrations, future studies are needed with a wider range of concentrations.
